# A rare case of giant intrascrotal rhabdomyosarcoma: A case report

**DOI:** 10.1016/j.ijscr.2025.112033

**Published:** 2025-10-07

**Authors:** Mejudin Kedir Abdella, Kaleab Habtemichael Gebreselassie, Shemsu Abraham Hussein, Woldie Jember Zewdie, Fedil Nuredin Abrar, Mesfin Assefa Tola

**Affiliations:** a Department of Urosurgery, Worabe Comprehensive Specialized Hospital, Worabe, Central Ethiopia Region, Ethiopia; b Department of Pathology, Wachemo University CMHS, Hosana, Central Ethiopia Region, Ethiopia; c Department of Pathology, Worabe Comprehensive Specialized Hospital, Worabe, Central Ethiopia Region, Ethiopia; d Onco Pathology Diagnostic Center, Addis Ababa, Ethiopia

**Keywords:** Rhabdomyosarcoma, Tumors, Scrotal, Testicular

## Abstract

**Introduction:**

Rhabdomyosarcoma can develop at a variety of sites, including the mesenchymal elements of the spermatic cord, epididymis, and testicular envelopes, resulting in the development of a painless scrotal mass.

We report this case because very few cases of intrascrotal rhabdomyosarcomas have been reported, especially in adolescents and adults; hence, we hope that this will contribute to the understanding, diagnosis, and treatment of the disease among academic societies.

**Case presentation:**

A 17-year-old male came with a 2-year history of progressive right scrotal swelling. There was a right testicular 20 × 15 cm mass.

Abdominopelvic CT-scan showed a huge, solid, and cystic enhancing right testicular mass.

Hematoxylin and eosin-stained histologic sections of the tumor showed highly cellular proliferation of pleomorphic spindle cells. Immunohistochemistry showed that the tumor cells are positive for desmin and myogenic.

The tumor mass was removed en bloc. The postoperative period was uneventful, and he was linked to the oncology department for subsequent evaluation and follow-up, but we could not find the patient's file at the oncology side to see his subsequent progress when we wrote this case report about one year later.

**Discussion:**

Intrascrotal rhabdomyosarcoma arises from mesenchymal components of the region and its diagnosis is based on a combination of clinical features, imaging findings, and histopathological features.

**Conclusion:**

Primary testicular rhabdomyosarcoma is rare but has a poor prognosis, particularly in adolescents and tumors larger than 10 cm; therefore, early diagnosis and aggressive surgical treatment reduces the incidence of local recurrence and may improve the overall survival.

## Highlights

• Intratesticular and paratesticular rhabdomyosarcomas can develop from mesenchymal elements of the spermatic cord, epididymis, and testicular envelopes.
• Para testicular rhabdomyosarcoma (RMS) is the most common non-germinal malignant tumor of the site.
• The diagnosis of intrascrotal rhabdomyosarcoma (RMS) is based on the combination of clinical features, imaging findings, and histopathologic features.
• Management of intrascrotal rhabdomyosarcoma encompasses surgery and chemotherapy, with or without radiation.

## 1 Introduction

Soft tissue sarcomas account for up to 3 % and 1 % of childhood and adult malignant tumors, respectively. Rhabdomyosarcoma is a malignant tumor of mesenchymal tissue origin thought to arise from or tend to differentiate to cells committed to a skeletal muscle lineage. It is the most common soft-tissue sarcoma in children and adolescents, with approximately 65 % of cases diagnosed in children less than six years of age and the remaining cases observed in the 10–18-year-old age group. Its incidence in adults is rare, accounting for only 3 % of soft tissue sarcomas, and there is a slight male preponderance. Rhabdomyosarcoma is the third most common extracranial childhood solid tumor after neuroblastoma and Wilms' tumor. The 2013 World Health Organization (WHO) classification of rhabdomyosarcoma includes four histological subtypes: alveolar, embryonal, pleomorphic, and sclerosing or spindle cell types, with the embryonal type representing approximately 70 % of all childhood rhabdomyosarcoma [[1], [2], [3]].

Rhabdomyosarcoma can occur at various sites. The orbit is the most common site for embryonal type in the head and neck region. Intratesticular and paratesticular rhabdomyosarcomas are rare, but they can develop from mesenchymal elements of the spermatic cord, epididymis, and testicular envelopes, resulting in the development of a painless scrotal mass. The tumor spreads mostly through lymphatics to the iliac and para-aortic nodes; however, hematogenous spread can also occur, most commonly to the lungs and liver.

Diagnosis of Para-and intratesticular rhabdomyosarcomas can be made with a high degree of clinical suspicion with the help of imaging diagnostic tests, biopsy, and immunohistochemistry [1,[3], [4], [5], [6]].

A case report from China revealed that a 21-year-old male diagnosed to have intrascrotal embryonal rhabdomyosarcoma after he presented with 1 1-year-long painless right testicular mass shows that the tumor can occur even in older individuals and supports our case of intrascrotal rhabdomyosarcoma in an adolescent patient. Another case report from Tanzania, on the other hand, revealed a diagnosis of intratesticular rhabdomyosarcoma in a 6-year-old male child, which is a common age for the tumor to occur, in contrast to our case, which we thought was among the rare occurrences [4,7].

Here we present a 17-year-old male patient diagnosed to have a giant intrascrotal rhabdomyosarcoma, which is very rare at this age and site.

We believe this report will contribute to describing the pathogenesis, clinical manifestations, diagnosis, treatment methods, and prognosis of intrascrotal rhabdomyosarcoma with the aim of improving the understanding of this rare disease.

This case report has been reported in line with the SCARE checklist [8].

## 2 Case presentation

### 2.1 History

A 17-year-old male came with a 2-year history of progressive right scrotal swelling. He visited a health facility 4 months ago and was diagnosed with epididymal TB after a scrotal ultrasound and FNAC, which showed granulomatous orchitis suggestive of TB. He was administered anti-TB for 3 months; however, the mass increased in size, and he lost significant weight. He presented to our hospital after 3 months of anti-TB treatment. He has no previous history of tuberculosis and no family history of the same illness.

### 2.2 Physical examination

**Fig. 1:**
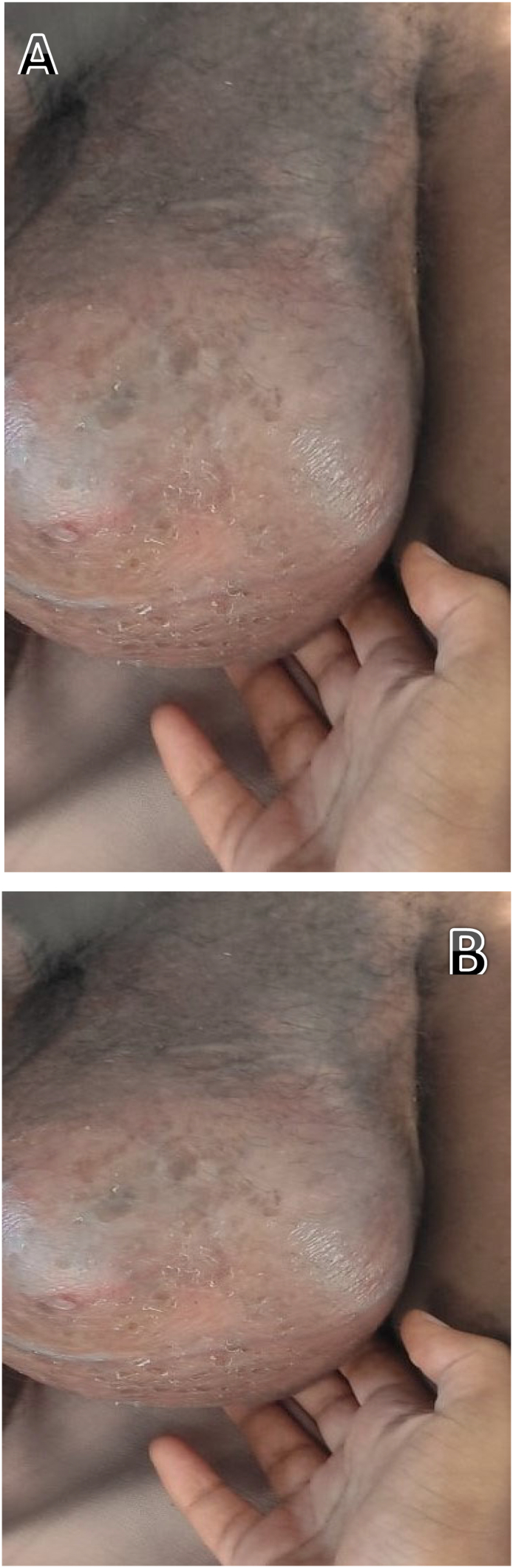
A&B-The gross physical appearance of right scrotal mass measuring about 20x15cm which had warm and cystic consistency.

Upon presentation, he looked chronically sick. His vital signs were within normal range except he had axillary temperature of 38.3. A right testicular 20 × 15 cm mass, with a warm and cystic consistency (Fig. 1A&B). The mass was adherent to the scrotal skin over the lower half, and there was a 3 × 3 mm ulceration with no discharge over the lower half of the anterior scrotum, likely the FNAC site. There was also a 1 × 1 cm right inguinal mobile lymph node.

### 2.3 Laboratory test findings

His white blood cell count was 8000 cells per microliter, Platelet count was 484,000 cells per microliter, hemoglobin level was 10.3 mg/dl, AFP level was 9.69 IU, BhCG level was 0.1 IU/L, and LDH -230 U/L/L.

### 2.4 Imaging findings

**Fig. 2:**
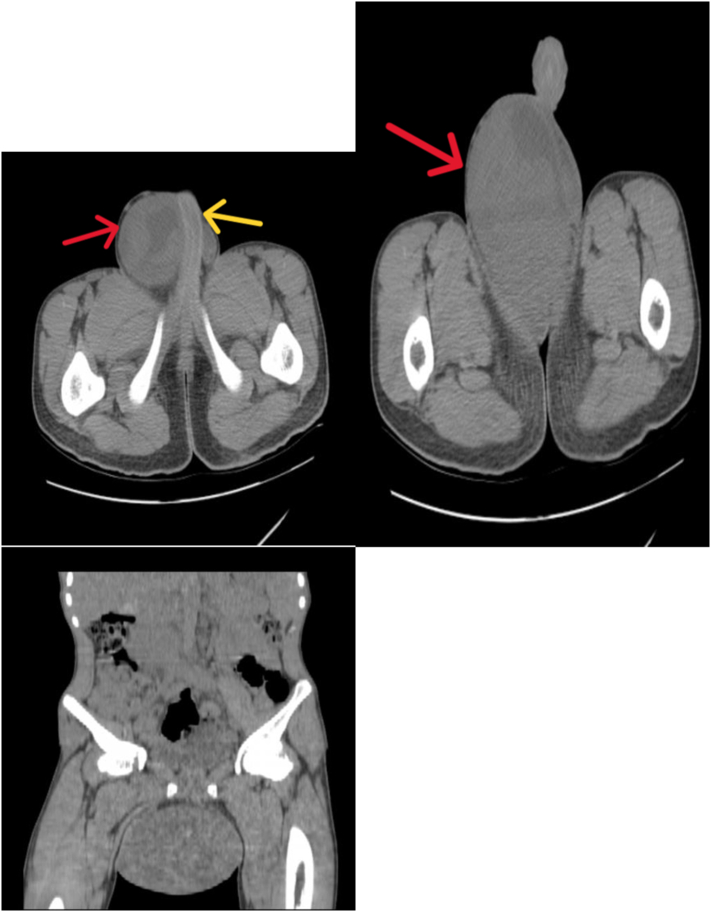
Pre-contrast CT scan images depicting a huge right scrotal mass (red arrows) pushing the penile shaft (yellow arrow) and extending distally between the thighs.

**Fig. 3:**
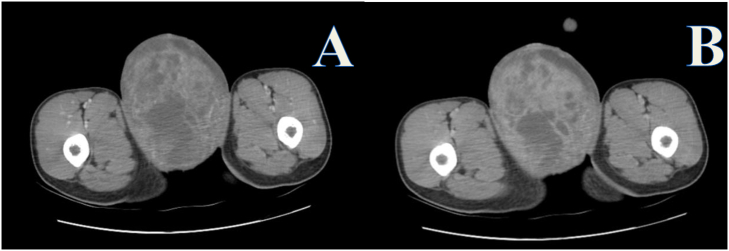
Contrast enhanced CT scan images with arterial (A) and portal venous (B) phases showing an heterogeneously enhancing huge scrotal mass with areas of necrosis.

**Fig. 4:**
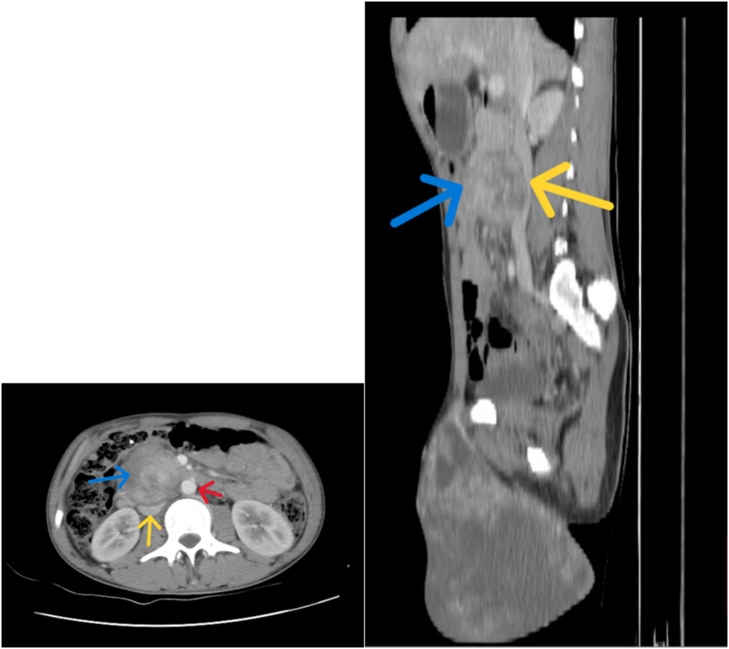
Contrast enhanced CT scan images of the abdomen depicting a heterogeneously enhancing retroperitoneal lymph node (blue arrows) pushing between the abdominal aorta (red arrow) and inferior vena cava (yellow arrows).

Abdominopelvic CT-scan showed a huge solid and cystic enhancing right testicular mass extending to the right inguinal canal with different-sized retroperitoneal lymphadenopathy, the largest being at the inter-aortocaval region with a size of 7 × 6 cm (Figs. 2A,B&C, 3A&B and 4A&B). Other abdominal organs look normal in appearance, and the chest x-ray is unremarkable.

### 2.5 Intraoperative findings

**Fig. 5:**
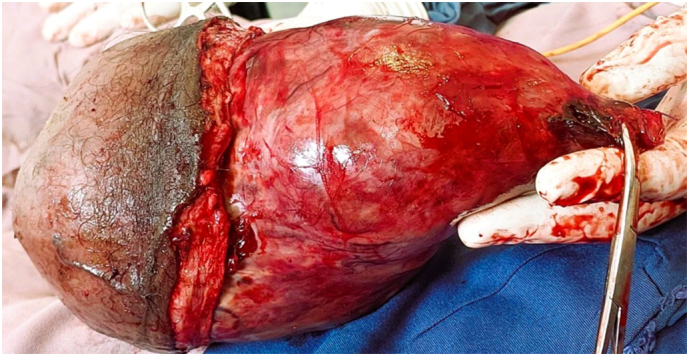
The gross appearance of the surgically removed right testicular mass with adherent lower scrotal skin.

A right inguinal incision was made, extending to the mid-scrotum. The spermatic cord was dissected free at the internal ring and ligated. The adherent scrotal skin was removed en bloc with approximately a 25 cm right scrotal solid and cystic mass with subdermal engorged vessels surrounding it, and the adherent lower half of the scrotal skin (Fig. 5) and the specimen was sent to the pathology department. The right testicle was not identified. The remaining scrotal skin was then reconstructed. The contralateral (left) testis and para-testicular structures were normal and not explored during surgery.

### 2.6 Histopathological evaluation findings

Gross: 22 × 17 × 15 cm, partly skin-covered tissue and a separate stitch-marked gray-brown tissue. On cut section solid and cystic gray-white to brown mass, with the latter measuring 5 × 5 cm. Three LNs were identified from separate gray-brown tissue fragments. No testicular parenchyma was identified, probably because it was completely replaced by the tumor.

**Fig. 6:**
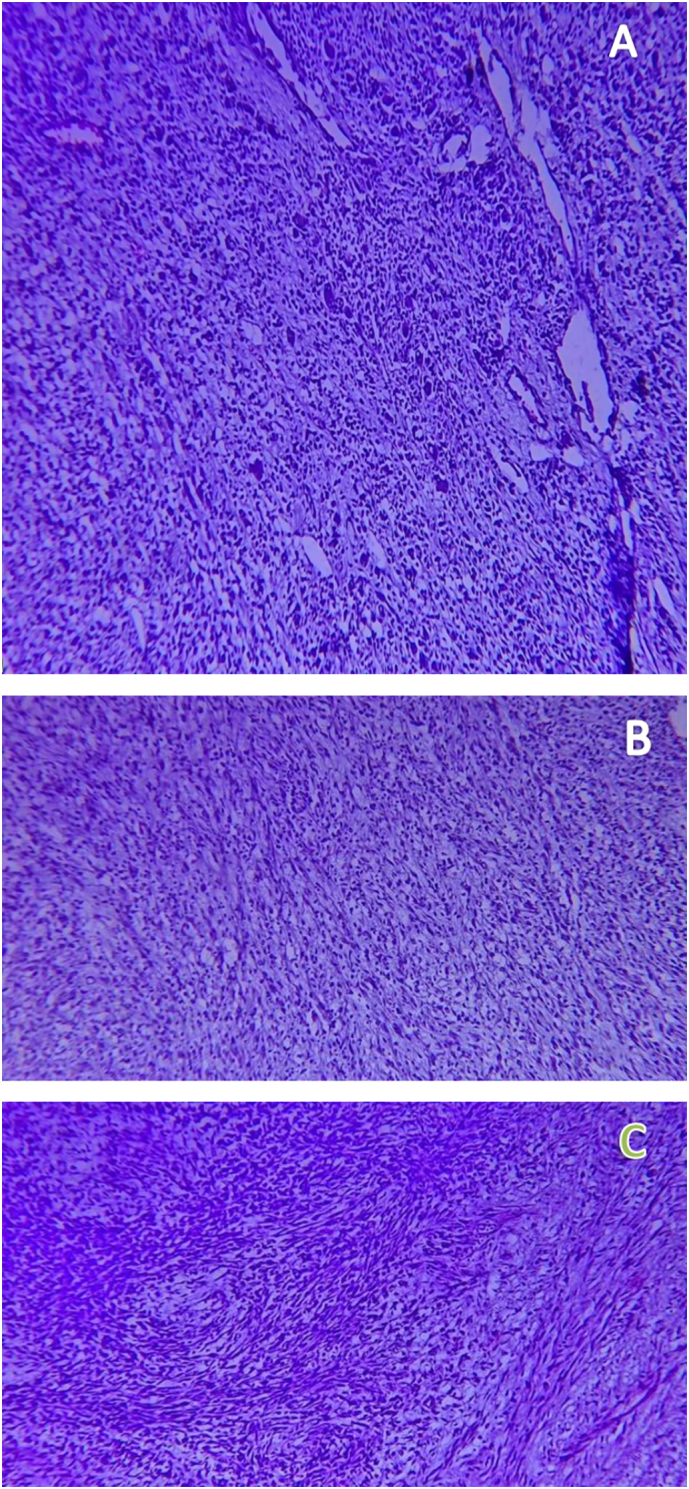
A,B&C-40× view of hematoxyline and eosin stained sections of different regions of the tumor exhibiting highly cellular proliferation of haphazardly arranged spindle cells embedded in a variably fibrous and myxoid stroma.

**Fig. 7:**
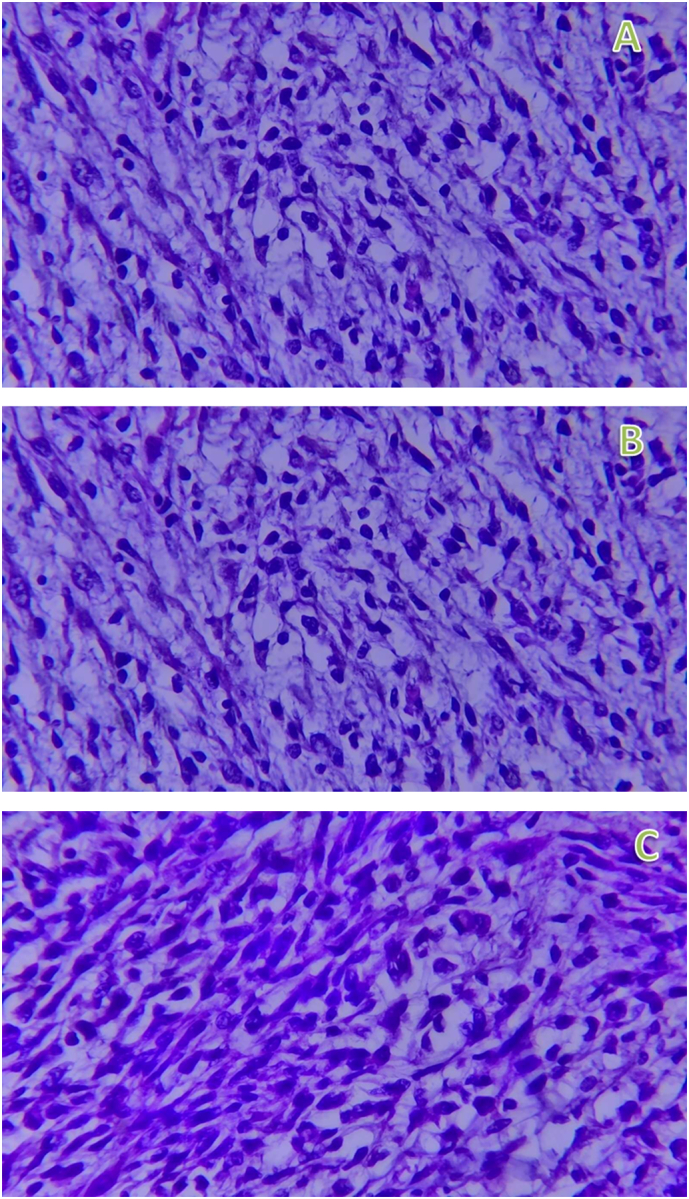
A,B&C-200× view of hematoxyline and eosin stained sections of different regions of the tumor exhibiting highly cellular proliferation of pleomorphic spindle cells arranged in vague fascicles.

**Fig. 8:**
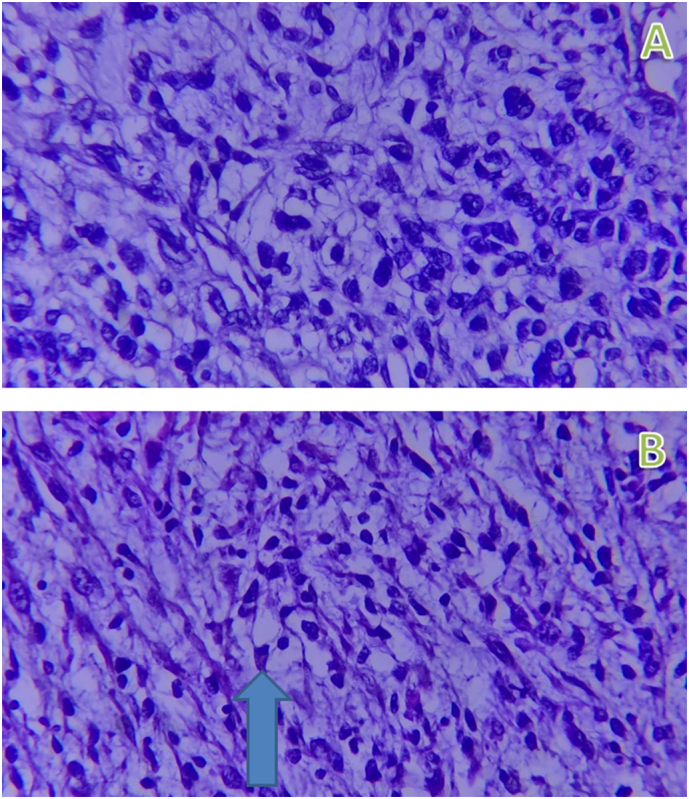
A&B-400× view of hematoxyline and eosin stained sections of different regions of the tumor exhibiting highly cellular proliferation of pleomorphic spindle cells arranged in vague fascicles with the arrow on figure-B indicating the rhabdoid cell exhibiting eccentric nucleus and abundant eosinophilic cytoplasm.

Microscopy: Hematoxylin and eosin-stained histologic sections of the tumor showed highly cellular proliferation predominantly composed of pleomorphic spindle cells with occasional polygonal cells having eccentric nuclei and eosinophilic cytoplasm arranged in sheets and haphazard fascicles (Fig. 6A,B&C (low power view), Fig. 7A,B&C (medium power view), Fig. 8A & B (high power view)). Sections from all three identified inguinal LNs disclosed reactive lymphoid hyperplasia with no tumor deposits.

**Fig. 9:**
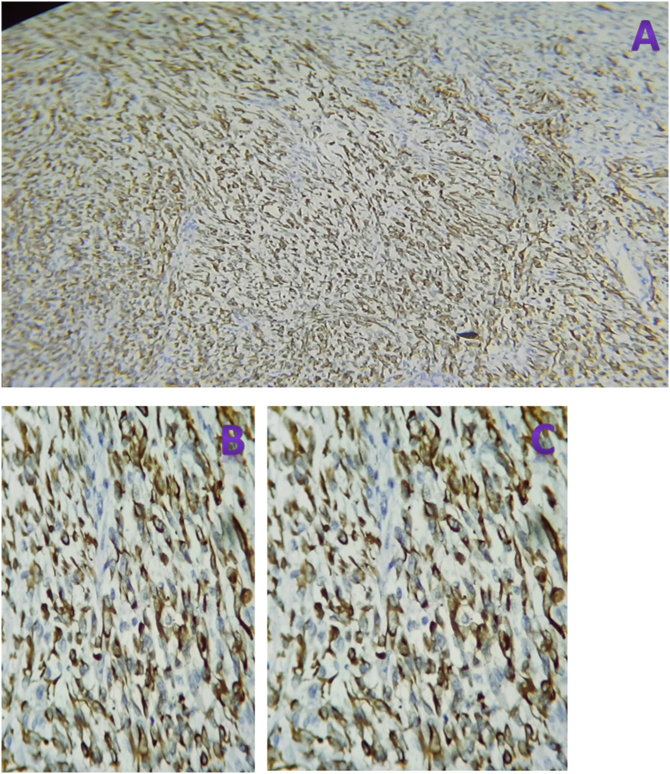
(A-40×,B-200× & C-400×) views of immunohistochemistry stained sections showing diffuse and strong cytoplasmic staining of the tumor cells for desmin.

**Fig. 10:**
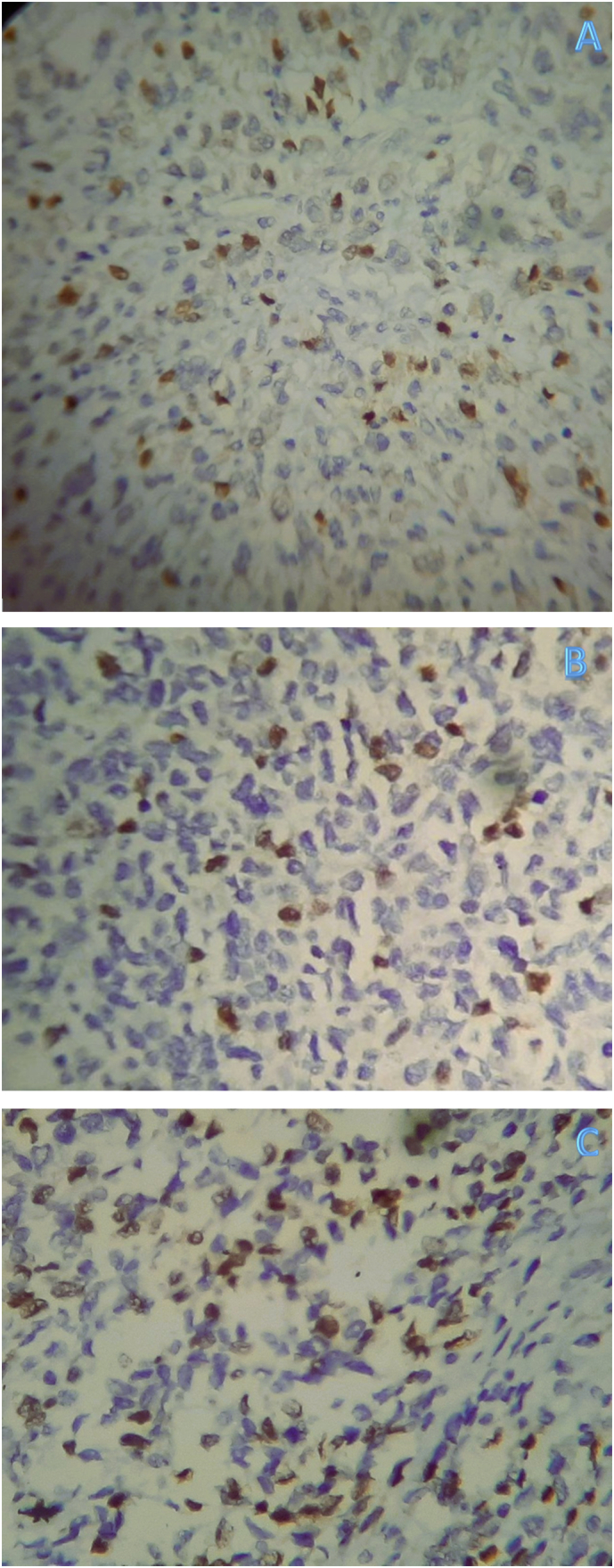
(A-40×,B-200× & C-400×) views of immunohistochemistry stained sections showing focal and strong nuclear staining of the tumor cells for myogenin.

Immunohistochemistry-stained sections show the tumor cells to be strongly and diffusely cytoplasmic positive for desmin (Fig. 9(A-40×,B-200× & C-400×)) and focally strongly nuclear positive for myogenin (Fig. 10(A-40×,B-200× & C-400×)). The tumor cells were negative for S100 and SOX10.

Other tumors can arise at this region and may clinically and radio logically mimic rhabdomyosarcoma especially the spindle cell type, such as leiomyosarcoma, liposarcoma, and fibrosarcoma, desmoplastic small round cell tumor, alveolar soft part sarcoma, and malignant fibrous histiocytoma.Thherefore, histopathologic and immunohistochemistry examination is usually required for definitive diagnosis.

This work has been reported in line with the SCARE criteria [9].

## 3 Discussion

There are three types of muscle cells in the body: smooth muscle cells in smooth muscles, skeletal muscle cells found in muscles that perform voluntary activities, and heart muscle cells found in the heart. Rhabdomyosarcoma is a type of soft tissue sarcoma that arises from immature mesenchyme cells, which normally become skeletal muscle. It is the most common soft-tissue tumor in children, but it is rare in adults. There are four recognized histopathological variants: embryonal, alveolar, spindle, and pleomorphic. Intrascrotal rhabdomyosarcoma can either be intratesticular or paratesticular, with the former being very rare and comprising less than 1 % of all testicular sarcomas, with a prevalence of 2 % of all testicular tumors, and the latter has an incidence of approximately 7 % of intrascrotal rhabdomyosarcoma. Para testicular rhabdomyosarcoma is the most common non-germinal malignant tumor at this site, and its clinical manifestations are non-specific, with B-HCG and alpha-fetoprotein (AFP) tumor markers usually not elevated. Embryonal rhabdomyosarcoma is common in adults under 30 years of age and usually presents as a large, painless, palpable mass (>5 cm). It can spread through the blood or lymphatic system, and the most common metastatic sites are the lungs, liver, and bone, with the para-aortic lymph nodes being involved in 26–43 % of cases. The diagnosis of intrascrotal rhabdomyosarcoma is based on the combination of clinical features, imaging findings (ultrasound, CT scan, and MRI), and histopathologic features. Ultrasonography is the first-line preferred imaging technique that can reliably distinguish between testicular and scrotal masses, with a sensitivity of >95 %. Both CT and MRI are usually used to evaluate the location, size, and metastasis of the tumor. At times, the specific diagnosis of rhabdomyosarcoma can be challenging with the above modalities alone.

[4,6,7,10]. The differential diagnosis of para-testicular rhabdomyosarcoma includes tumors such as leiomyosarcoma, liposarcoma, and fibrosarcoma, desmoplastic small round cell tumor, alveolar soft part sarcoma, and malignant fibrous histiocytoma. Because these tumors lack specific diagnostic clinical and imaging features, confirmatory diagnosis relies on postoperative histopathologic and immunohistochemistry evaluation and rhabdomyosarcoma particularly the spindle cell type is differentiated from other spindle cell sarcomas by their positivity to one or more muscle-specific markers, such as desmin, muscle-specific actin, MyoD1, myoglobin, and/or myogenin, especially myogenin is very specific and sensitive for rhabdomyosarcoma [3,5,11,12].

Our patient was a 17-year-old male patient presented with painless right scrotal swelling. He underwent surgery with a urosurgeon, and the preoperative diagnosis was a malignant tumor. However, histopathologic and immunohistochemistry evaluations revealed that it was a spindle cell type rhabdomyosarcoma. Its exact origin was difficult to confirm owing to its large size and the absence of a normal testicle.

Sarcoma and tuberculosis are common diseases affecting health worldwide, and diagnostic confusion between these entities may arise, especially in tuberculosis-endemic areas, due to factors such as early nonspecific clinical presentations, some spindle cells may resemble granuloma-forming epitheloid cells on fine needle aspiration cytology evaluation, and some sarcomas may contain a reactive granulomatous inflammation component [13]. Therefore, multiple diagnostic modalities and proper follow-up are advised in order not to miss patients.

Our patient was initially diagnosed with testicular tuberculosis by FNAC and started on anti-tuberculosis medication, but the mass progressively increased in size while he was taking the medication.

A study conducted in South Africa showed that 45 (77 %) of the 58 children with rhabdomyosarcomas had the embryonal subtype and 12 (21 %) had the alveolar type, with the median age at diagnosis for all types being 5.3 years. A cancer incidence estimate in Ethiopia in 2015 revealed that soft tissue sarcoma accounted for 5 % of all childhood cancers. A recent study conducted in Ethiopia showed that two-thirds (66 %) of patients with rhabdomyosarcoma were diagnosed under 5 years of age, and more than 50 % of pediatric rhabdomyosarcoma cases occurred in the head and neck region, with the orbit being the most common site of involvement. The study also showed a slightly more common occurrence in males, with the embryonic subtype being the most frequent type, accounting for 53.7 % of the cases, followed by alveolar subtypes.

The presenting age of our patient is among the less common groups, which shows that rhabdomyosarcoma should be considered in patients with painless scrotal masses. Management of intrascrotal Rhabdomyosarcoma remains controversial due to its rarity and limited cases in the literature. In general, a comprehensive treatment strategy that encompasses surgery and chemotherapy, with or without radiation, has been developed [1,10,14].

Our patient's tumor mass was removed en block with the adherent part of the scrotal skin through an inguinal incision, and the postoperative period was uneventful; he was linked to the oncology department for subsequent evaluation and follow-up, but we could not find the patient's file at the oncology side to see his subsequent progress when we write this case report after about one year.

Because only a few studies of intrascrotal rhabdomyosarcomas have been reported, especially in adolescents and adults, we hope that this case report will contribute to the understanding, diagnosis, and treatment of the disease among academic societies.

## 4 Conclusion

Primary testicular rhabdomyosarcoma is rare and has a poor prognosis, particularly in adolescents and tumors larger than 10 cm. Diagnosis is mostly done following histological examination of the resected specimen because ultrasound findings are usually non-specific, and testicular tumor markers are not elevated. Radical orchidectomy is feasible even in the presence of a giant scrotal mass; hence, early diagnosis and radical orchidectomy improve the prognosis. Long-term follow-up is recommended, especially in adult patients, to monitor for relapse.

## Consent for publication

- Parental consent (for minors):

Written informed consent was obtained from the patient's parents/legal guardian for publication and any accompanying images. A copy of the written consent is available for review by the Editor-in-Chief of this journal on request.

## Ethical approval

No ethical approval was sought by the authors as the study did not involve any animal or human experiments, and our hospital is a community hospital with limited research activities so far, which made finding an organized institutional review board difficult.

## Guarantor

Woldie Jember Zewdie.

## Funding

All authors declare that this work did not receive any funding from any sector.

## Author contribution

Woldie Jember Zewdie: Conceptualization; Visualization; writing – original draft; and editing.

Mejudin Kedir Abdella: Data curation; Writing – review and editing.

Kaleab Habtemichael Gebreselassie: Visualization; Writing – review and editing.

Fedil Nuredin Abrar: Data curation; Writing – review and editing.

Mesfin Assefa Tola: Supervision; Visualization; Writing – review and editing.

Shemsu Abraham Hussein: Data curation; Writing – review and editing.

## Declaration of competing interest

The authors declare that they have no competing financial interests or personal relationships that could have influenced the work reported in this paper.
